# Controllable and fast quantum-information transfer between distant nodes in two-dimensional networks

**DOI:** 10.1038/s41598-016-0016-1

**Published:** 2016-12-05

**Authors:** Zhi-Rong Zhong

**Affiliations:** Department of Physics, Fuzhou University, Fuzhou, 350002 P. R. China

## Abstract

We construct shortcuts to adiabatic passage to achieve controllable and fast quantum-information transfer (QIT) between arbitrary two distant nodes in a two-dimensional (2D) quantum network. Through suitable designing of time-dependent Rabi frequencies, we show that perfect QIT between arbitrary two distant nodes can be rapidly achieved. Numerical simulations demonstrate that the proposal is robust to the decoherence caused by atomic spontaneous emission and cavity photon leakage. Additionally, the proposed scheme is also insensitive to the variations of the experimental parameters. Thus, the proposed scheme provides a new perspective on robust quantum information processing in 2D quantum networks.

## Introduction

In quantum information and quantum computation, one of the essential ingredients is the realization of controllable and fast quantum-information transfer (QIT) between arbitrary remote nodes in a quantum network. In recent years, the QIT has been accomplished by several approaches, i.e., the resonant *π* pulses, composite pulses, stimulated Raman adiabatic passage (STIRAP), and their variants^1–3^. Although the resonant *π* pulse technique can fast transfer quantum information^1^, its highly sensitivity to the deviations of pulse areas restricts its extensive application in quantum information processing. The adiabatic passage techniques^2,3^ are robust versus variations of the experimental parameters while they usually need a long operation time. Thus the decoherence, which is one of the main obstacles in quantum information and quantum computation, would strongly affect the dynamics of the system, furthermore, may lead to the schemes become useless. The “shortcuts to adiabaticity technique”, which combines the advantages of resonant *π* pulses and adiabatic techniques, has been considered as a promising venue to achieve fast and high-fidelity QIT, and has attracted much attention in recent years^4–15^. In view of shortcuts to adiabaticity, Chen and Muga^6,7^ have successfully performed fast population transfer in three-level atom systems via applying the opposite variation tendency in the time-dependent laser pulse. After that, the shortcuts to adiabaticity technique has been extended from one-atom system to two-or multi-atom system^12–15^.

The scalability is still another obstacles in accomplishing the quantum information and quantum computation under current cavity quantum electrodynamics technology. The emergence of coupled cavity system^16^ which can overcome the scalability and meet the requirement of kinds of quantum tasks, i.e., simulation of quantum many-body phenomena^17–23^, performing remote quantum information transfer^24–26^, entanglement generation^27–35^ and quantum gate operations between two distant nodes^36,37^. All such works typically focus on the cases of either two-site or one-dimensional (1D) coupled cavity arrays. Extending such researches to more complex coupled cavity arrays (i.e., two-dimensional (2D) or three-dimensional (3D)) is more significance for quantum computation. There have been several studies considering the 2D coupled cavity arrays, which have respectively considered the realization of the fractional quantum Hall system^38^ and 2D one-way quantum computation^39^. Recently, we have proposed protocols to realize the coherent coupling of multiple atoms^40^ and to realize two-qubits unconventional geometric phase gates in a 2D coupled cavity array^41^.

The quantum Zeno effect is an interesting phenomenon in quantum mechanics and has been demonstrated in many experiments^42–45^. It has been shown that a system can actually evolve away from its initial state, but still remain in the Zeno subspace defined by the measurements via frequently projecting onto a multidimensional subspace, which is known as quantum Zeno dynamics (QZD)^46–48^. In general, if a system is governed by Hamiltonian *H*_*K*_ = *H*_obs_ + *KH*_meas_, where *H*_obs_ is the Hamiltonian of the subsystem to be investigated, *H*_meas_ is an additional interaction Hamiltonian performing the “measurement”, and *K* is a coupling constant. In a strong coupling limit *K* → ∞, the whole system will remain in the same Zeno subspace, and is governed by the evolution operator defined as $U(t)=\exp (\,-\,it{\sum }_{n}K{E}_{n}{P}_{n}+{P}_{n}{H}_{{\rm{obs}}}{P}_{n})$, with *P*_*n*_ being the eigenvalue projection of *H*_meas_ with eigenvalues *E*_*n*_ (*H*_meas_ = ∑_*n*_ *E*_*n*_ *P*_*n*_).

Motivated by the space division of QZD, in this paper, we construct shortcuts to adiabatic passage to achieve controllable and fast QIT between arbitrary two nodes in a 2D quantum network. Through suitably designing the time-dependent Rabi frequencies, we can controllably and fast transfer quantum-information between arbitrary two distant nodes in one-step. The distinguished advantages of the proposal are: (i) information can be controllably transferred between arbitrary two nodes; (ii) the time to accomplish the task is shorter than that in conventional adiabatic passage technique; (iii) it is robust against the parameters fluctuations and the decoherence caused by atomic spontaneous emission and cavity photon leakage. Thus it provides a new perspective on robust quantum information processing in 2D quantum networks in the future.

## The theoretical model and the construction of a shortcut passage

**Figure 1:**
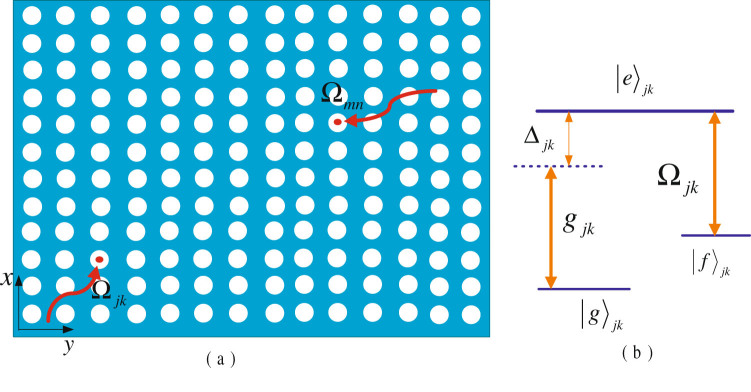
(**a**) Schematic diagram of a two-dimensional (2D) array of coupled cavities. Each node contains a Λ-type three-level atom and can respectively couple to their neighboring ones through the *x* and *y* directions with intercavity photon hopping. (**b**) The atom level scheme. The transition of the *jk*th atom |*g*〉_*jk*_ ↔ |*e*〉_*jk*_ is coupled to the cavity mode with detuning Δ_*jk*_, the corresponding coupling rate is *g*_*jk*_. The transition |*f*〉_*jk*_ ↔ |*e*〉_*jk*_ of the *jk*th atom is resonantly driven by a classical laser field, and the corresponding Rabi frequencies are Ω_*jk*_.

we consider a 2D (*N* × *N*) coupled cavity array, as shown in Fig. 1(a). Each cavity (denoted by *jk*) respectively couples to their neighboring ones through the *x* and *y* directions with intercavity photon hopping. Each cavity contains a Λ-type atom. The atoms have two ground states (labeled as ${|g\rangle }_{jk}$ and ${|f\rangle }_{jk}$) and one excited state (labeled as ${|e\rangle }_{jk}$), as shown in Fig. 1(b). The ${|g\rangle }_{jk}\leftrightarrow {|e\rangle }_{jk}$ transition of atom couples to the corresponding cavity mode with coupling rate *g*_*jk*_ and detuning Δ_*jk*_. The ${|f\rangle }_{jk}\leftrightarrow {|e\rangle }_{jk}$ transition of atom is resonantly driven by a classical field with Rabi frequency Ω_*jk*_ (*j*, *k* ∈ 1, …, *N*). In the interaction picture, the Hamiltonian for the system is (*ħ* = 1)

1 $$H={H}_{1}+{H}_{2},$$

with

2 $${H}_{1}=\sum _{j,k=1}^{N}{[{g}_{jk}{a}_{jk}|e\rangle }_{jk}{\langle g|{e}^{i{{\rm{\Delta }}}_{jk}t}+{{\rm{\Omega }}}_{jk}|e\rangle }_{jk}\langle f|+{\rm{H}}{\rm{.c}}\mathrm{.]},$$

and

3 $${H}_{2}=\sum _{j,k=1}^{N}[v{a}_{jk}{a}_{j+\mathrm{1,}k}^{+}+v{a}_{jk}{a}_{j,k+1}^{+}+{\rm{H}}{\rm{.c}}\mathrm{.]},$$

where *a*_*jk*_ $({a}_{jk}^{+})$ denotes the annihilation (creation) operator for the *jk*th cavity, and *v* is the hopping rate of photons between neighboring cavities. We adopt periodic boundary conditions *a*_*j*1_ = *a*_*jN*_ and *a*_1*k*_ = *a*_*Nk*_ by introducing the nonlocal bosonic modes *c*_*mn*_, and diagonalize the Hamiltonian *H*_2_ via the Fourier transform: ${a}_{jk}=\frac{1}{N}{\sum }_{m,n}^{N}\exp [-\,i(\frac{2\pi jm}{N}+\frac{2\pi kn}{N})]{c}_{mn}$. Thus we can rewrite the Hamiltonian *H*_1_ and *H*_2_ as

4 $${H}_{1}=\sum _{j,k=1}^{N}[{{\rm{\Omega }}}_{jk}|e{\rangle }_{jk}{\langle f|+\sum _{m,n}\frac{{g}_{jk}}{N}{e}^{-i(\frac{2\pi jm}{N}+\frac{2\pi kn}{N})}{e}^{i{{\rm{\Delta }}}_{jk}t}|e\rangle }_{jk}\langle g|{c}_{mn}+{\rm{H}}{\rm{.c}}{\rm{.}}],$$

and

5 $${H}_{2}=\sum _{m,n}^{N}{\omega }_{mn}{c}_{mn}^{+}{c}_{mn}+{\rm{H}}{\rm{.c}}{\rm{.}},$$

where ${\omega }_{mn}=2v(\cos \,\frac{2\pi n}{N}+\,\cos \,\frac{2\pi m}{N})$ (*n *= 0,1,2, …, *N* − 1, *m* = 0, 1, 2, …, *N* − 1). We now go into a new frame by defining *H*_2_ as a free Hamiltonian, and obtain the interaction Hamiltonian for the whole system as

6 $${H}_{1}^{^{\prime} }=\sum _{j,k}^{N}\{{{\rm{\Omega }}}_{jk}|e{\rangle }_{jk}\langle f|+\sum _{m,n}[\frac{{g}_{jk}}{N}{e}^{-i(\frac{2\pi jm}{N}+\frac{2\pi kn}{N})}{e}^{i({{\rm{\Delta }}}_{jk}-{\omega }_{mn})t}{c}_{mn}|e{\rangle }_{jk}\langle g|+{\rm{H}}{\rm{.c}}{\rm{.}}]\}.$$

In order to achieve the information transfer between arbitrary two distant nodes. We first assume that the atom to receive information is in the |*g*〉_*NN*_ state (in general, we assume that the atom in node NN), the atoms in other nodes are all in the state |*f*_*jk*_〉 (*jk* ≠ *NN*). Assume that the information to be transferred is loaded in the node 11 (i.e., the atom in node 11 is initially in the |*f*〉_11_ state), and the cavity modes are all in the vacuum state. Next, we apply two laser fields with Rabi frequency Ω_*jk*_ to these two nodes (node 11 and NN). Thus, the Hamiltonian in Eq. (6) reduces to

7 $${H}_{1}^{^{\prime} }=\sum _{j,k=\mathrm{11,}NN}\{{{\rm{\Omega }}}_{jk}|e{\rangle }_{jk}\langle f|+\sum _{m,n}[\frac{{g}_{jk}}{N}{e}^{-i(\frac{2\pi jm}{N}+\frac{2\pi kn}{N})}{e}^{i({{\rm{\Delta }}}_{jk}-{\omega }_{mn})t}{c}_{m,n}|e{\rangle }_{jk}\langle g|+{\rm{H}}{\rm{.c}}{\rm{.}}]\}\mathrm{.}$$

Generally, the accurate dynamics evolution governed by the above Hamiltonian is complicated, but there still can be simplified in some regimes. If the atomic transition frequencies are set equal to one of the frequencies of nonlocal bosonic modes, i.e, appropriately adjusting the detuning to satisfy Δ_11_ = Δ_*NN*_ = *ω*_*pq*_, the Hamiltonian in Eq. (7) becomes

8 $${H}_{{\rm{eff}}}=\sum _{j,k=\mathrm{11,}NN}\{{[{{\rm{\Omega }}}_{jk}|e\rangle }_{jk}{\langle f|+{g}_{p,q}{c}_{p,q}|e\rangle }_{jk}\langle g|+{\rm{H}}{\rm{.c}}.]+\sum _{m,n\ne p,q}\sum _{jk=\mathrm{11,}NN}[\frac{{g}_{jk}}{N}{e}^{-i(\frac{2\pi jm}{N}+\frac{2\pi kn}{N})}\\ \quad \quad \quad \times {e}^{i({{\rm{\Delta }}}_{jk}-{\omega }_{mn})t}{c}_{m,n}|e{\rangle }_{jk}\langle g|+{\rm{H}}{\rm{.c}}{\rm{.}}]\},$$

with ${g}_{p,q}=\frac{{g}_{jk}}{N}{e}^{-i(\frac{2\pi jp}{N}+\frac{2\pi kq}{N})}$. The first part in the above Hamiltonian describes the resonant interaction between atoms and the nonlocal bosonic mode as well as the laser fields, while the second term represents the dispersive interaction between the atoms and the nonresonant normal modes. Under the condition ${{\rm{\Delta }}}_{jk}-{\omega }_{mn}\gg \frac{{g}_{jk}}{N}{e}^{-i(\frac{2\pi jm}{m}+\frac{2\pi kn}{n})}$, the interaction of the atoms with the nonresonant normal modes can be neglected, the Hamiltonian reads

$${H}_{{\rm{eff}}}={H}_{{\rm{1eff}}}+{H}_{{\rm{2eff}}},$$

where

$${H}_{{\rm{1eff}}}=\sum _{j,k=\mathrm{11,}NN}{{\rm{\Omega }}}_{jk}|e{\rangle }_{jk}\langle f|+{\rm{H}}{\rm{.c}}{\rm{.}},$$

and

9 $${H}_{{\rm{2eff}}}=\sum _{j,k=\mathrm{11,}NN}{g}_{p,q}{c}_{p,q}|e{\rangle }_{jk}\langle g|+{\rm{H}}{\rm{.c}}{\rm{.}}$$

The above Hamiltonian *H*_eff_ shows that, the atoms can resonantly interact with the nonlocal bosonic mode *c*_*p*,*q*_, it means that the atoms resonantly interact with all the cavities simultaneously. Assume the system is initially in the state |Φ_0_〉 = |*f*_11_〉|*g*_*NN*_〉|0〉 (i.e., atoms in the node 11 and node NN are in the states |*f*〉 and |*g*〉, respectively, and the bosonic mode *C*_*pq*_ is in the vacuum state), the whole system evolves in the subspace spanned by $\{|{{\rm{\Phi }}}_{1}\rangle$ $={|f\rangle }_{11}{|g\rangle }_{NN}|0\rangle$, $|{\Phi }_{2}\rangle ={|e\rangle }_{11}{|g\rangle }_{NN}|0\rangle$, $|{\Phi }_{3}\rangle ={|g\rangle }_{11}{|g\rangle }_{NN}|1\rangle$, $|{\Phi }_{4}\rangle ={|g\rangle }_{11}{|e\rangle }_{NN}|0\rangle$, $|{\Phi }_{5}\rangle ={|g\rangle }_{11}{|f\rangle }_{NN}|0\rangle \}$. In the proposed model, the interaction between atoms and nonlocal bosonic mode plays the role of continuous measurements on the interaction between atoms and the classical fields. Thus, we concentrate on the dynamical evolution of three new computation bases. In light of QZD, the eigenvalues of *H*_2eff_ are *E*_1_ = 0, *E*_2_ = −*g*, *E*_3_ = −*g* (we here assume *g*_*pq*_ = *g*). Thus, the corresponding eigenvectors of Hamiltonian *H*_2eff_ are

10 $$\begin{array}{rcl}|{{\rm{\Psi }}}_{1}\rangle  & = & \frac{1}{\sqrt{2}}(\,-\,|{{\rm{\Phi }}}_{2}\rangle +|{{\rm{\Phi }}}_{4}\rangle ),\,|{{\rm{\Psi }}}_{2}\rangle =\frac{1}{2}(|{{\rm{\Phi }}}_{2}\rangle -\sqrt{2}|{{\rm{\Phi }}}_{3}\rangle +|{{\rm{\Phi }}}_{4}\rangle ),\\ |{{\rm{\Psi }}}_{3}\rangle  & = & \frac{1}{2}(|{{\rm{\Phi }}}_{2}\rangle +\sqrt{2}|{{\rm{\Phi }}}_{3}\rangle +|{{\rm{\Phi }}}_{4}\rangle)\end{array}.$$

Next, we rewrite the Hamiltonian in Eq. (9) with the eigenvectors of *H*_2eff_,

$${H}_{{\rm{eff}}}^{^{\prime} }={H}_{{\rm{1eff}}}^{^{\prime} }+{H}_{{\rm{2eff}}}^{^{\prime} },$$

where

$${H}_{{\rm{1eff}}}^{^{\prime} }=\sum _{i=1}^{3}{E}_{i}|{{\rm{\Psi }}}_{i}\rangle \langle {{\rm{\Psi }}}_{i}|,$$

and

11 $$\begin{array}{rcl}{H}_{{\rm{2eff}}}^{^{\prime} } & = & \frac{{{\rm{\Omega }}}_{11}(t)}{\sqrt{2}}|{{\rm{\Psi }}}_{1}\rangle \langle {{\rm{\Phi }}}_{1}|+\frac{{{\rm{\Omega }}}_{11}(t)}{2}(|{{\rm{\Psi }}}_{2}\rangle +|{{\rm{\Psi }}}_{3}\rangle )\langle {{\rm{\Phi }}}_{1}|+\frac{{{\rm{\Omega }}}_{NN}(t)}{\sqrt{2}}|{{\rm{\Psi }}}_{1}\rangle \langle {{\rm{\Phi }}}_{1}|\\  &  & +\frac{{{\rm{\Omega }}}_{NN}(t)}{2}(|{{\rm{\Psi }}}_{2}\rangle +|{{\rm{\Psi }}}_{3}\rangle )\langle {{\rm{\Phi }}}_{1}|+{\rm{H}}{\rm{.c}}.\end{array}$$

It is obvious that there are four nonzero energy eigenvalues ±Ω_11_(*t*) and ±Ω_*NN*_(*t*) for the Hamiltonian ${H}_{{\rm{2eff}}}^{^{\prime} }$. Defining ${H}_{{\rm{1eff}}}^{^{\prime} }$ as a free Hamiltonian, and performing the unitary transformation $U={e}^{-i{H}_{{{\rm{1eff}}}^{t}}^{^{\prime} }}$ under condition ${H}_{{\rm{2eff}}}^{^{\prime} }\gg {H}_{{\rm{1eff}}}^{^{\prime} }$, we obtain

12 $$\begin{array}{rcl} {H}_{{\rm{2eff}}}^{^{\prime} } & = & \frac{{{\rm{\Omega }}}_{11}(t)}{\sqrt{2}}|{{\rm{\Psi }}}_{1}\rangle \langle {{\rm{\Phi }}}_{1}|+\frac{{{\rm{\Omega }}}_{11}(t)}{2}({e}^{i\sqrt{2}gt}|{{\rm{\Psi }}}_{2}\rangle +{e}^{-i\sqrt{2}gt}|{{\rm{\Psi }}}_{3}\rangle )\langle {{\rm{\Phi }}}_{1}|+\frac{{{\rm{\Omega }}}_{NN}(t)}{\sqrt{2}}|{{\rm{\Psi }}}_{1}\rangle \langle {{\rm{\Phi }}}_{5}|\\  &  & +\frac{{{\rm{\Omega }}}_{NN}(t)}{2}({e}^{i\sqrt{2}gt}|{{\rm{\Psi }}}_{2}\rangle +{e}^{-i\sqrt{2}gt}|{{\rm{\Psi }}}_{3}\rangle )\langle {{\rm{\Phi }}}_{5}|+{\rm{H}}{\rm{.c}}.\end{array}$$

Therefore, setting $\sqrt{2}g\gg {{\rm{\Omega }}}_{11}(t),{{\rm{\Omega }}}_{NN}(t)$, the condition ${H}_{{\rm{2eff}}}^{^{\prime} }\gg {H}_{{\rm{1eff}}}^{^{\prime} }$ and the Zeno condition *K* → ∞ are satisfied. Under the rotating-wave approximation, we have a new Hamiltonian

13 $${H}_{{\rm{2eff}}}^{^{\prime\prime} }=\frac{{{\rm{\Omega }}}_{11}(t)}{\sqrt{2}}|{{\rm{\Psi }}}_{1}\rangle \langle {{\rm{\Phi }}}_{1}|+\frac{{{\rm{\Omega }}}_{NN}(t)}{\sqrt{2}}|{{\rm{\Psi }}}_{1}\rangle \langle {{\rm{\Phi }}}_{5}|+{\rm{H}}{\rm{.c}}{\rm{.}}$$

Thus the Hilbert subspace splits into three invariant Zeno subspaces *H*_p0_ = {|Ψ_1_〉,|Φ_1_〉,|Φ_5_〉}, *H*_p1_ = {|Ψ_2_〉}, *H*_p2_ = {|Ψ_3_〉}. The system can be divided into three subsystems, *S*_1_ = {|Ψ_1_〉,|Φ_1_〉,|Φ_5_〉}, *S*_2_ = {|Ψ_2_〉,|Φ_1_〉,|Φ_5_〉}, *S*_3_ = {|Ψ_3_〉,|Φ_1_〉,|Φ_5_〉}. Note that the interaction between the states in subsystems *S*_2_ and *S*_3_ is far weaker than that in subsystem *S*_1_, thus this weak interaction can be neglected. Then the system can be considered as a three-level atom system with two ground states |Φ_1_〉,|Φ_5_〉 and an excited state |Ψ_1_〉. The Hamiltonian for STIRAP reads

14 $${H}_{{s}_{2}}=\frac{1}{\sqrt{2}}\left(\begin{array}{ccc} 0 & {{\rm{\Omega }}}_{11}(t) & 0\\ {{\rm{\Omega }}}_{11}(t) & 0 & {{\rm{\Omega }}}_{NN}(t)\\ 0 & {{\rm{\Omega }}}_{NN}(t) & 0\end{array}\right).$$

The instantaneous eigenvalues are ${\chi }_{0}=\mathrm{0,}{\chi }_{\pm }=\pm \,\sqrt{{{\rm{\Omega }}}_{11}^{2}+{{\rm{\Omega }}}_{NN}^{2}}/\sqrt{2}$, with the corresponding eigenstates are

15 $$|{{\rm{\Phi }}}_{0}(t)\rangle = \left(\begin{array}{c} \cos \,\theta \\ 0\\ \sin \,\theta\end{array}\right),|{{\rm{\Phi }}}_{\pm }(t)\rangle =\left(\begin{array}{c}-\,\sin \,\theta \\ \pm 1\\ \cos \,\theta\end{array}\right).$$

Here $\theta =\arctan ({{\rm{\Omega }}}_{11}(t)/{{\rm{\Omega }}}_{NN}(t))$. Thus, if the adiabatic condition $|\dot{\theta }|\ll \chi /\sqrt{2}$ is fulfilled, QIT from initial state |Φ_1_〉 to target state |Φ_5_〉 is achieved adiabatically along the dark state |Φ_0_〉. However, the time to accomplished this task is long.

Next we introduce how to construct shortcuts to fast transfer quantum-information by using the dynamics of invariant based inverse engineering^7^. Here, we first introduce an invariant Hermitian operator ${I}_{{s}_{2}}(t)$, which satisfies the Schröodinger equation $i\partial {I}_{{s}_{2}}(t)/\partial t=[{H}_{{s}_{2}}(t),{I}_{{s}_{2}}(t)]$ ^49^, for ${H}_{{s}_{2}}(t)$ possesses the SU(2) dynamical symmetry. And ${I}_{{s}_{2}}(t)$ is given by

16 $${I}_{{s}_{2}}(t)=\frac{\chi }{\sqrt{2}}\left(\begin{array}{ccc} 0 & \cos \,\gamma \,\sin \,\beta  & -i\,\sin \,\gamma \\ \cos \,\gamma \,\sin \,\beta  & 0 & \cos \,\gamma \,\cos \,\beta \\ i\,\sin \,\gamma  & \cos \,\gamma \,\cos \,\beta  & 0\end{array}\right).$$

Here *γ* and *β* are the time-dependent auxiliary parameters and satisfy the following equations,

17 $$\dot{\gamma }=\frac{1}{\sqrt{2}}[{{\rm{\Omega }}}_{11}(t)\cos \,\beta -{{\rm{\Omega }}}_{NN}(t)\sin \,\beta ],\,\dot{\beta }=\frac{1}{\sqrt{2}}\,\tan \,\gamma [{{\rm{\Omega }}}_{NN}(t)\cos \,\beta +{{\rm{\Omega }}}_{11}(t)\sin \,\beta ],$$

where the dot represents a time derivative. By inversely deriving from Eq. (17), the explicit expressions of Ω_11_(*t*) and Ω_*NN*_(*t*) are as follows:

18 $${{\rm{\Omega }}}_{11}(t)=\sqrt{2}[\dot{\beta }\,\cot \,\gamma \,\sin \,\beta -\dot{\gamma }\,\cos \,\beta ],\,{{\rm{\Omega }}}_{NN}(t)=\sqrt{2}[\dot{\beta }\,\cot \,\gamma \,\cos \,\beta -\dot{\gamma }\,\sin \,\beta \mathrm{].}$$

The eigenstates of the invariant *I*_*s*2_(*t*) are

19 $$|{\Phi }_{0}^{^{\prime} }(t)\rangle = \left(\begin{array}{c} \cos \,\gamma \,\cos \,\beta \\ -i\,\sin \,\gamma \\ -\,\cos \,\gamma \,\sin \,\beta\end{array}\right),|{{\rm{\Phi }}}_{\pm }^{^{\prime} }(t)\rangle = \left(\begin{array}{c}\sin \,\gamma \,\cos \,\beta \pm i\,\sin \,\beta \\ i\,\cos \,\gamma \\ -\,\sin \,\gamma \,\sin \,\beta \pm i\,\cos \,\beta\end{array}\right),$$

corresponding to the eigenvalues *λ*_0_ = 0 and *λ*_±_ = ±1, respectively. Based on the Lewis-Riesenfeld theory^50^, the solution of the Schröodinger equation with respect to the instantaneous eigenstates of ${I}_{{s}_{2}}(t)$ is a superposition of orthonormal dynamical modes, $|{\rm{\Psi }}(t)\rangle ={\sum }_{n}{C}_{n}{e}^{i{\alpha }_{n}}|{{\rm{\Phi }}}_{n}(t)\rangle$, where *C*_*n*_ is a time-independent amplitude and *α*_*n*_ is the Lewis-Riesenfeld phase and obeys the form,

20 $${\alpha }_{n}({t}_{f})=\frac{1}{\hslash }{\int }_{0}^{{t}_{f}}\langle {{\rm{\Phi }}}_{n}(t)|[i\frac{\partial }{\partial t}-{H}_{s2}(t)]|{{\rm{\Phi }}}_{n}(t)\rangle d{t}^{\text{'}}.$$

In the proposal, *α*_0_ = 0, and

21 $$\alpha (\pm )=\mp \,\frac{1}{\hslash }{\int }_{0}^{{t}_{f}}[\dot{\beta }\,\sin \,\gamma -\frac{1}{2}({{\rm{\Omega }}}_{11}\,\sin \,\beta +{{\rm{\Omega }}}_{NN}\,\cos \,\beta )\cos \,\gamma ]d{t}^{\text{'}}.$$

In order to get the target state |Φ_5_〉 along the invariant eigenstate ${|{\rm{\Phi }}}_{0}^{\text{'}}(t)\rangle$, we suitably choose the feasible parameters *γ*(*t*) and *β*(*t*),

22 $$\gamma (t)=\xi ,\beta (t)=\pi t\mathrm{/2}{t}_{f},$$

where *ξ* is a small value, which satisfies (sin*ξ*)^−1^ = 4 *M*(*M* = 1, 2, 3, …) for a high fidelity of the target state. And we obtain

23 $${{\rm{\Omega }}}_{11}(t)=\pi t/(\sqrt{2}{t}_{f})\cot \,\xi \,\sin (\pi t\mathrm{/2}{t}_{f}),\,{{\rm{\Omega }}}_{NN}(t)=\pi t/(\sqrt{2}{t}_{f})\cot \,\xi \,\cos (\pi t\mathrm{/2}{t}_{f}\mathrm{).}$$

Once the Rabi frequencies are specially designed, the fast QIT from initial state to the target state in subsystem *S*_1_ will be implemented.

## Results

**Figure 2:**
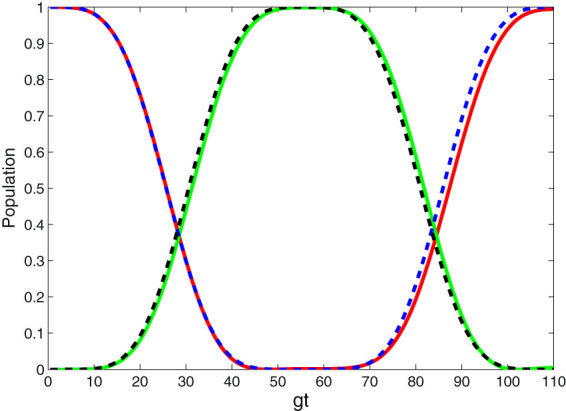
The comparison between the information transfer governed by the total Hamiltonian *H*_*I*_ and that governed by the effective Hamiltonian ${H}_{{S}_{2}}$ when *v* = 2.0 *g*, *gt*_*f*_ = 50, $\xi =\arcsin \mathrm{(0.25)}$. The red-solid (green-solid) and blue-dashed (black-dashed) lines describe the time evolution of the population of state $|{\psi }_{1}\rangle =|{f}_{11}{g}_{22}{0}_{11}{0}_{12}{0}_{21}{0}_{22}\rangle$($|{\psi }_{8}\rangle =|{g}_{11}{f}_{22}{0}_{11}{0}_{12}{0}_{21}{0}_{22}\rangle$) and state $|{{\rm{\Phi }}}_{1}\rangle =|{f}_{11}{g}_{22}{0}_{pq}\rangle$ ($|{{\rm{\Phi }}}_{5}\rangle =|{g}_{11}{f}_{22}{0}_{pq}\rangle$) governed by the total Hamiltonian and effective Hamiltonian, respectively.

To confirm the validity of all our above derivation, we first numerically simulate the dynamics governed by the derived effective Hamiltonian in Eq. (14), and compare it to the dynamics governed by the total Hamiltonian in Eq. (1). Note that the numerical computation we performed using the python package Qutip^51^. The validity of the model is numerically simulated by taking the evolution of the population *P* = |〈*ψ*|*ψ*(*t*)〉|^2^ of the proposed state |*ψ*〉. We consider the case with *N* = 2, and set the parameters in the following way: *v* = 2.0 *g*, *gt*_*f*_ = 50, $\xi =\arcsin \mathrm{(0.25)}$ (the Zeno condition $\sqrt{2}g\gg {{\rm{\Omega }}}_{jk}(t)$ can be satisfied very well). For the total Hamiltonian, a new subspace is spanned by $\{|{\psi }_{1}\rangle =|f{\rangle }_{11}|g{\rangle }_{22}|0{\rangle }_{11}|0{\rangle }_{12}|0{\rangle }_{21}|0{\rangle }_{22}$, ${\psi }_{2}\rangle =|e{\rangle }_{11}|g{\rangle }_{22}|0{\rangle }_{11}|0{\rangle }_{12}|0{\rangle }_{21}|0{\rangle }_{22}$, ${\psi }_{3}\rangle =|g{\rangle }_{11}|e{\rangle }_{22}|0{\rangle }_{11}|0{\rangle }_{12}|0{\rangle }_{21}|0{\rangle }_{22}$, ${\psi }_{4}\rangle =|g{\rangle }_{11}|g{\rangle }_{22}|1{\rangle }_{11}|0{\rangle }_{12}|0{\rangle }_{21}|0{\rangle }_{22}$, ${\psi }_{5}\rangle =|g{\rangle }_{11}|g{\rangle }_{22}|0{\rangle }_{11}|1{\rangle }_{12}|0{\rangle }_{21}|0{\rangle }_{22}$, ${\psi }_{6}\rangle =|g{\rangle }_{11}|g{\rangle }_{22}|0{\rangle }_{11}|0{\rangle }_{12}|1{\rangle }_{21}|0{\rangle }_{22}$, ${\psi }_{7}\rangle =|g{\rangle }_{11}|g{\rangle }_{22}|0{\rangle }_{11}|0{\rangle }_{12}|0{\rangle }_{21}|1{\rangle }_{22}$, ${\psi }_{8}\rangle =|g{\rangle }_{11}|f{\rangle }_{22}|0{\rangle }_{11}|0{\rangle }_{12}|0{\rangle }_{21}|0{\rangle }_{22}\}$. Thus, if the system is initially in one of these basics, the system will evolve in this subspace. In Fig. 2, the red-solid (green-solid) and blue-dashed (black-dashed) lines describe the time evolution of the population of state |*ψ*_1_〉 = |*f*_11_ *g*_22_ 0_11_ 0_12_ 0_21_ 0_22_〉 (|*ψ*_8_〉 = |*f*_22_ 0_11_ 0_12_ 0_21_ 0_22_〉) and state |Φ_1_〉 = |*f*_11_ *g*_22_ 0_*pq*_〉 (|Φ_5_〉 = |*g*_11_ *f*_22_ 0_*pq*_〉) governed by the total Hamiltonian and effective Hamiltonian, respectively. It is obvious that the approximations adopted during the deriving of the effective Hamiltonian are valid, since the two curves described by the total Hamiltonian and effective Hamiltonian are nearly coincided, and their deviation is small enough as soon as the parameters are fixed.

**Figure 3:**
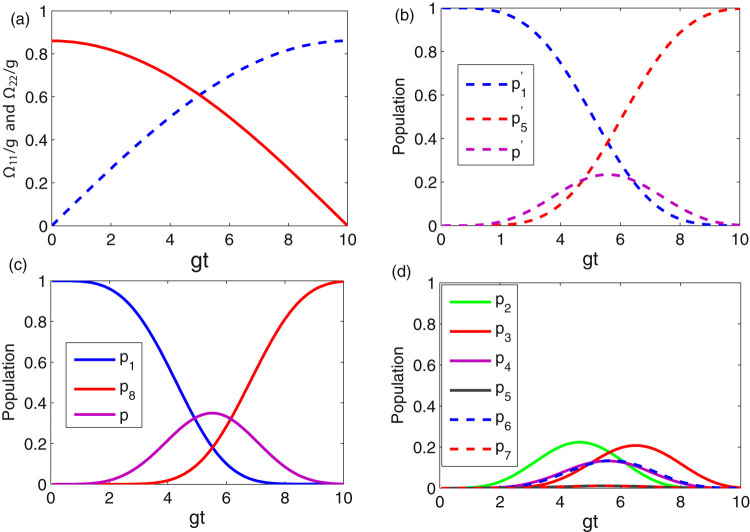
(**a**) The time dependence of the laser fields Ω_11_(*t*) and Ω_*NN*_(*t*) when *λt*_*f*_ = 10, *ξ* = 0.25, and *v* = 2.0 *g*. (**b**) The time evolution of populations governed by the effective Hamiltonian ${H}_{{S}_{2}}$ for the states |Φ_1_〉, |Φ_5_〉, and |Ψ_1_〉 when *λt*_*f*_ = 10, *ξ* = 0.25, and *v* = 2.0 *g*. (**c**) The time evolution of populations governed by the total Hamiltonian *H*_*I*_ for the states |*ψ*_1_〉, |*ψ*_8_〉, and $|\psi \rangle =\mathrm{1/}\sqrt{2}(|{\psi }_{2}\rangle +|{\psi }_{3}\rangle )$ when *λt*_*f*_ = 10, *ξ* = 0.25, and *v* = 2.0 *g*. (**d**) The time evolution of populations governed by the total Hamiltonian *H*_*I*_ for the states |*ψ*_2_〉, |*ψ*_3_〉, |*ψ*_3_〉, |*ψ*_5_〉, |*ψ*_6_〉, |*ψ*_7_〉 when *λt*_*f*_ = 10, *ξ* = 0.25, and *v* = 2.0 *g*.

Next, we show how the operation time is shorten when considering the shortcuts to adiabatic passage. We first numerically simulate the time dependence of the Rabi frequencies for the atoms in Fig. 3(a) when *gt*_*f*_ = 10, the other parameters are set the same as those in Fig. 2. As seen from Fig. 3(a), the maximum value of Ω_*jk*_/*g* is 0.83, which satisfies the conditions mentioned above (the Zeno condition $\sqrt{2}g\gg {{\rm{\Omega }}}_{jk}(t)$ (*jk* = 11, *NN*). In Fig. 3(b), we plot the time evolution of the populations of states |Φ_1_〉 = |*f*〉_11_|*g*〉_22_|0〉_*pq*_ (blue-dash line), |Φ_5_〉 = |*g*〉_11_|*f*〉_22_|0〉_*pq*_ (red-dash line), and $|{{\rm{\Psi }}}_{1}\rangle =\mathrm{1/}\sqrt{2}{(|e\rangle }_{11}|g{\rangle }_{22}\mathrm{|0}{\rangle }_{pq}+|g{\rangle }_{11}|e{\rangle }_{22}\mathrm{|0}{\rangle }_{pq})$ (magenta-dash line) under effective Hamiltonian in Eq. (14). Figure 3(b) shows that a perfect and fast quantum-information transfer from the initial state |Φ_1_〉 to the target state |Φ_5_〉 can be achieved after reselecting the optimal value of *ξ*. Notice that the population of excited state |Ψ_1_〉 is less than 0.25 during the interaction. Thus, we can draw a conclusion that the effective model can be considered as a three-level single-atom model^7^, as the optimal value of *ξ* for the whole system faultlessly satisfy the condition. In Fig. 3(c), we plot the time evolution of the population of states |*ψ*_1_〉 = |*f*〉_11_|*g*〉_22_|0〉_11_|0〉_12_|0〉_21_|0〉_22_ (blue-solid line), |*ψ*_8_〉 = |*g*〉_11_|*f*〉_22_|0〉_11_|0〉_12_|0〉_21_|0〉_22_ (red-solid line), and $|\psi \rangle =\mathrm{1/}\sqrt{2}{(|g\rangle }_{11}|e{\rangle }_{22}\mathrm{|0}{\rangle }_{11}\mathrm{|0}{\rangle }_{12}\mathrm{|0}{\rangle }_{21}\mathrm{|0}{\rangle }_{22}+|e{\rangle }_{11}|f{\rangle }_{22}\mathrm{|0}{\rangle }_{11}\mathrm{|0}{\rangle }_{12}\mathrm{|0}{\rangle }_{21}\mathrm{|0}{\rangle }_{22})$ (magenta-solid line) under the total Hamiltonian in Eq. (1). Also, a perfect and fast QIT from the initial state |*ψ*_1_〉 to the target state |*ψ*_8_〉 can be achieved at time *t*_*f*_. Compared to the effective Hamiltonian model, the population of excited state governed by total Hamiltonian is larger than that governed by the effective Hamiltonian. The reason for this can be explained as follow: during the operation, the intermediate states (i.e., |*g*〉_11_|*g*〉_22_|1〉_11_|0〉_12_|0〉_21_|0〉_22_, |*g*〉_11_|*g*〉_22_|0〉_11_|1〉_12_|0〉_21_|0〉_22_, etc.) can be slightly populated, the whole system cannot be faultlessly considered as a three-level single-atom model, and the optimal value of *ξ* for the whole system will not faultlessly satisfy the condition ${(\sin \xi )}^{-}\mathrm{1=4}\,M$. In order to get more insight to dynamic of the system governed by the total Hamiltonian, we plot the population of states |*ψ*_2_〉 to |*ψ*_7_〉 versus time in Fig. 3(d). From Fig. 3(d), we can see that all the populations of these states are smaller than 0.25, especially, the probabilities to find a photon in nodes 12 and 21 are less than 0.012. We can draw a conclusion that the system can be approximately considered as a three-level atom system, although the specific procedures has small differences between the two dynamics. Thus, the information can be fast and perfect transferred between arbitrary two distant nodes under current condition.

**Figure 4:**
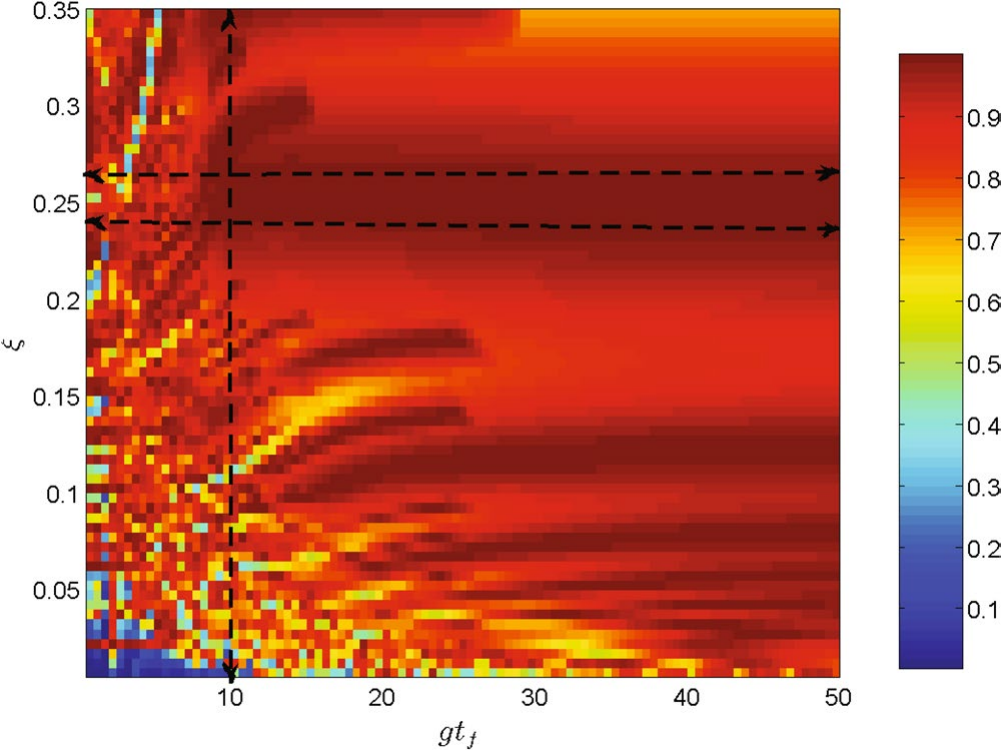
The fidelity of the target state |*ψ*_8_〉 versus the value of *ξ* and the interaction time *gt*_*f*_ governed by the total Hamiltonian *H*_*I*_ when *v* = 2.0 *g*. The fastest time to get the high fidelity of the target state is *t*_*f*_ = 10/*g* when $\xi \simeq 0.25$ (from 0.235 to 0.265).

As shown in Fig. (3), the proposal can be nearly treated as an adiabatic process which is insensitive to the fluctuations of parameters, such as the amplitude of the laser pulses Ω_*jk*_, the coupling constant *g* and the parameter *ξ*. Thus, we can choose a sets of parameters to obtain high fidelity and fast QIT. In Fig. 4, we plot the fidelity of the target state |*ψ*_8_〉 versus the value of *ξ* and the interaction time *gt*_*f*_ governed by the total Hamiltonian *H*_*I*_ when *v* = 2.0 *g*. The fidelity for the target state is defined as $F=\langle {\psi }_{8}|\rho (t)|{\psi }_{8}\rangle$, where *ρ*(*t*) is the density operator of the system at the time *t*_*f*_ by solving the equation $\dot{\rho }=i[{H}_{I},\rho ]$. As seen from Fig. 4, when *gt*_*f*_ = 10, the optimal value of *ξ* for the highest fidelity (fidelity $\simeq 1$) of the state |*ψ*_8_〉 is from 0.235 to 0.265. The reason for this can be expressed as: the proposal is a adiabatic passage, thus it is robust versus variations in the experimental parameters. However, when the parameters are no longer approximately satisfied by the condition ${(\sin \xi )}^{-1}=4\,M$ (*M* = 1, 2, 3, …), the fidelity will show an extreme fluctuation. Figure 4 also shows that it is hardly to get high fidelity when *gt*_*f*_ < 10. Thus, in the proposed scheme, the fastest time to get the target state is *t*_*f*_ = 10/*g*. Therefore, it is much faster than the general adiabatic process.

## Discussion

**Figure 5:**
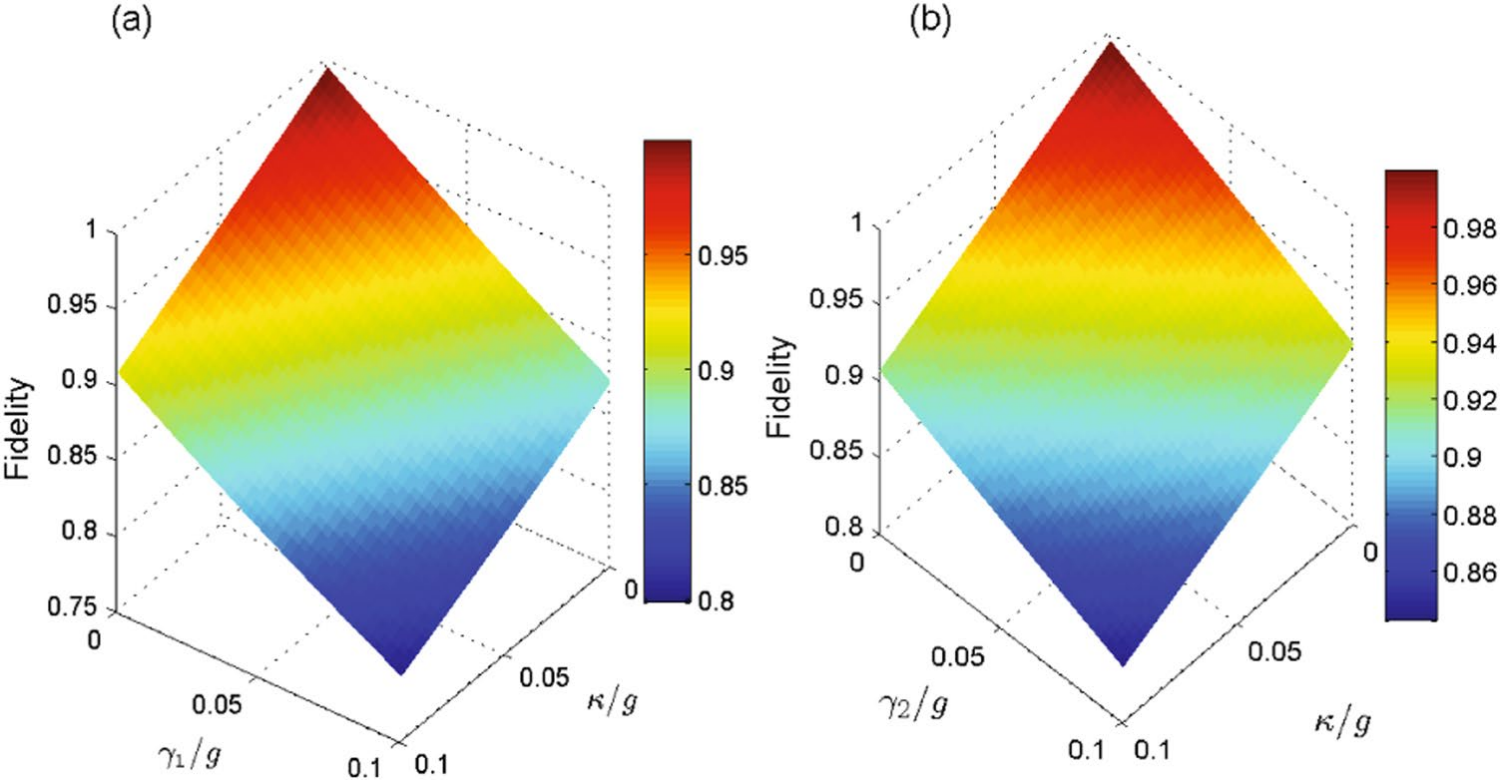
(**a**) The fidelity of the target state |*ψ*_8_〉 versus versus the ratios *γ*_1_/*g* and *κ*/*g*. (**b**) The fidelity of the target state |*ψ*_8_〉 versus versus the ratios *γ*_2_/*g* and *κ*/*g*. The others parameters are set as *ξ* = 0.25 and *gt*_*f*_ = 10. The fidelity is still about 79.8% (83.3%) when *κ* = *γ*_1_ = 0.1 *g* in Fig. 5(a) (*κ* = *γ*_2_ = 0.1 *g* in Fig. 5(b)).

It is necessary to discuss the influence of decoherence caused by atomic spontaneous emission and cavity photon leakage of the system. In the current model, the master equation of the whole system can be expressed by the Lindblad form^52^,

24 $$\begin{array}{rcl}\dot{\rho } & = & -\,i[{H}_{I},\rho ]+\sum _{ls=11}^{22}\frac{{\kappa }_{ls}}{2}\mathrm{(2}{a}_{ls}\rho {a}_{ls}^{+}-{a}_{ls}^{+}{a}_{ls}\rho -\rho {a}_{ls}^{+}{a}_{ls})\\  &  & +\sum _{jk=\mathrm{11,22}}\frac{{\gamma }_{jk}^{eg}}{2}\mathrm{(2}{\sigma }_{ee}^{jk}\rho {\sigma }_{ee}^{jk}-{\sigma }_{ee}^{jk}\rho -\rho {\sigma }_{ee}^{jk})\\  &  & +\sum _{jk\mathrm{=11,22}}\frac{{\gamma }_{jk}^{ef}}{2}\mathrm{(2}{\sigma }_{ee}^{jk}\rho {\sigma }_{ee}^{jk}-{\sigma }_{ee}^{jk}\rho -\rho {\sigma }_{ee}^{jk}),\end{array}$$

where *κ*_*ls*_ denotes the decay rate of cavity, ${\gamma }_{jk}^{eg}$ and ${\gamma }_{jk}^{ef}$ represent the atomic decay from level |*e*_*jk*_〉 to |*g*_*jk*_〉 and |*e*_*jk*_〉 to |*f*_*jk*_〉, respectively. For simplicity, we assume *κ*_*ls*_ = *κ* (*ls* = 11, 12, 21, 22), ${\gamma }_{jk}^{eg}={\gamma }_{1}$ and ${\gamma }_{jk}^{ef}={\gamma }_{2}$ (*jk* = 11, 22). The fidelity of the target state versus the ratios *κ* and *γ*_1_ (*κ* and *γ*_2_) is shown in Fig. 5(a,b) when *ξ* = 0.25 and *gt*_*f*_ = 10. As seen from Fig. 5, the fidelity decreases slowly with the increasing of cavity decay and atomic spontaneous emission. Figure 5 shows that the fidelity is still about 79.8% (83.3%) when *κ* = *γ*_1_ = 0.1 *g* (*κ* = *γ*_2_ = 0.1 *g*). Therefore, we can draw a conclusion that the proposal is robust against the spontaneous emission and cavity photon leakage.

Finally, let us give a brief analysis of the experimental feasibility for this scheme. The proposal can be realized in solid-state qubit trapped in a 2D array of superconducting cavity system. In this system, the superconducting cavity can be strongly coupled to the solid-state qubits such as Cooper pair boxes (CPB), and the corresponding microwave photons have small loss rates. As reported in ref. 53, the coupling strength in the interaction between CPBs and the circuit cavities is *g*˜2*π* × 50 MHZ, the corresponding photon lifetime is *T*_*c*_ ~ 20 × 10^−6^ s, the dephasing time of the spin state is *T*_*a*_˜1 × 10^−6^ s. Thus, the required time for transferring the quantum-information, in principle, is *T*~3.2 × 10^−8^ s, which is much shorter than *T*_*c*_ and *T*_*a*_. The proposed idea can also be used for large-scale arrays cavities in photonic crystals, in which the achievable parameters are predicted to be (*g*,*κ*,*γ*) = 2*π* × (2.5 × 10^3^, 0.4, 1.6) MHz^54^. As shown above, the required time for achieving the task is smaller than photon coherence time and the atom dephasing time. In recent experiments, a set of cavity quantum electrodynamics parameters (*g*, *κ*, *γ*) = 2*π* × (7.6, 2.8, 3.0) MHz is available in an optical cavity^55–57^. Thus, based on the recent cavity QED technique or the technique to be improved soon, the proposal might be realizable in the future.

In conclusion, we have proposed a promising scheme to construct shortcuts to adiabatic passage to achieve controllable and fast quantum-information transfer between arbitrary two nodes in 2D quantum networks. The proposal has several advantages. The first one is that information can be controllably transferred between arbitrary two nodes, which is the basic of quantum computation. Secondly, the operation time is shorter than that in conventional adiabatic passage technique. Third, the proposed scheme is robust against the parameter fluctuations and the decoherence caused by atomic spontaneous emission and cavity photon leakage. These are very benefit to the suppression of decoherence effect. The scheme provides a new perspective on robust quantum information processing in 2D quantum networks. In principle, the proposal can be realized in solid-state qubit trapped in a 2D array of superconducting cavity system or in large-scale arrays cavities in photonic crystals. Moreover, the proposed scheme can be extended to 3D coupled cavity system.
